# Revealing the dynamics of saffron growth: Optimizing corm size and planting depth for increased yield synergies

**DOI:** 10.1371/journal.pone.0303264

**Published:** 2024-05-17

**Authors:** Ghulam Sarwar, Tauseef Anwar, Huma Qureshi, Muhammad Younus, Muhammad Waqar Hassan, Muhammad Sajid-ur-Rehman, Faizan Khalid, Muhammad Ishaq, Bushra Ahmed Alhammad, Mahmoud F. Seleiman

**Affiliations:** 1 Department of Botany, The Islamia University of Bahawalpur, Bahawalpur, Pakistan; 2 Department of Botany, University of Chakwal, Chakwal, Pakistan; 3 Department of Pharmacognosy, The Islamia University of Bahawalpur, Bahawalpur, Pakistan; 4 Department of Entomology, The Islamia University of Bahawalpur, Bahawalpur, Pakistan; 5 Biology Department, College of Science and Humanity Studies, Prince Sattam Bin Abdulaziz University, Riyadh, Saudi Arabia; 6 Department of Plant Production, College of Food and Agriculture Sciences, King Saud University, Riyadh, Saudi Arabia; KGUT: Graduate University of Advanced Technology, ISLAMIC REPUBLIC OF IRAN

## Abstract

Saffron, the "golden spice" derived from *Crocus sativus* L., is renowned for its richness in secondary metabolites such as crocin and safranal, contributing to its unique properties. Facing challenges like decreasing global production, optimizing cultivation techniques becomes imperative for enhanced yields. Although the impact of factors like planting density, planting depth, spacing, and corm size on saffron growth has been studied, the interaction between corm size and planting depth remains underexplored. This study systematically investigates the interactive effects of corm size and planting depth on saffron growth and yield, providing evidence-based guidelines for optimizing cultivation. A factorial experiment, employing a completely randomized design, was conducted to assess the influence of corm size (05-10g, 10.1-15g, 15.1-20g) and planting depth (10cm, 15cm, 20cm) on saffron yield. Uniform-sized corms were obtained, and a suitable soil mixture was prepared for cultivation. Morphological and agronomic parameters were measured, and statistical analyses were performed using ANOVA and Tukey’s HSD test. The study revealed that planting depth significantly affected saffron emergence. The corms sown under 15cm depth showed 100% emergence regardless of corm size (either 05-10g, 10.1-15g, 15.1-20g) followed by 10cm depth corms. Corm dry weight exhibited a complex interaction, where larger corms benefited from deeper planting, while intermediate-sized corms thrived at shallower depths. Similar patterns were observed in shoot fresh weight and dry weight. Specifically, the largest corm size (t3, 15.1-20g) produced the greatest fresh-weight biomass at the deepest planting depth of 20cm (T3), while intermediate-sized corms (t2, 10.1-15g) were superior at the shallowest 10cm depth (T1). The total plant biomass demonstrated that larger corms excelled in deeper planting, while intermediate-sized corms were optimal at moderate depths. This research highlights the intricate interplay between corm size and planting depth in influencing saffron growth. Larger corms generally promote higher biomass, but the interaction with planting depth is crucial. Understanding these dynamics can aid farmers in tailoring cultivation practices for optimal saffron yields. The study emphasizes the need for a coordinated approach to corm selection and depth placement, providing valuable insights for sustainable saffron production and economic growth.

## Introduction

Saffron, also known as golden spice or red gold or ‘Kesar’ is one of the most expensive spices in the world by weight. It is obtained from the stigmas of *Crocus sativus* L. which belongs to the family ‘Iridaceae’ [1]. Saffron is thought to have originated in ancient civilizations including Greco-Roman, Egyptian, and Persian cultures around 4000 years ago. The 9th-century Arab conquests brought saffron to North Africa. Later, the Moorish invasion introduced saffron to Spain and wider Europe, especially France through the 11th-13th century Crusades [2]. Saffron contains over 150 secondary metabolites that provide its characteristic properties. The main compounds include crocin, safranal, and picrocrocin [3]. Crocin is a group of glycoside derivatives from the carotenoid crocetin. Crocin content is an important index to measure the quality of saffron. The characteristic aroma of saffron is due to another bioactive compound called safranal [2]. In addition to their use as a spice, crocin and safranal have also shown antioxidant, anti-cancer, anti-depressant, and other pharmacological effects in various studies [4–6].

For the past few years, there has been a constant decrease in global saffron production owing to issues like increasing salinity, drought, pests and diseases, postharvest handling, and poor-quality corms, pressing the urgent need to find ways to optimize the yield [2]. As saffron is the world’s costliest spice by weight, enhancing low yields through optimized cultivation techniques presents valuable economic potential. Various previous studies have found the role of planting density [7, 8], planting depth [9–11], spacing [11], and corm size [12] in improving the growth and yield of saffron. Sharifi *et al*. [8] identified corm size (8±1 g) and planting depth (15±2 cm) as variables that significantly contribute to saffron emergence rates and growth patterns. Additionally, Mollafilabi *et al*. [13] studied three corm weight categories (6–8, 8–10, and >10 g) and found all measured traits were significantly affected by increasing size. Banhangi *et al*. [7] also reported reduced total dry matter as planting depths increased from 10 to 20cm. However, the interaction of corm size and planting depth on the growth and yield of saffron in the current study area largely remains unexplored.

Therefore, our recent controlled analysis systematically examined how different corm sizes interacted with different planting depths to impact saffron growth patterns and yields. Results delineated actionable evidence-based guidelines for ideal coordinated corm selection and depth placement to improve saffron growth. Findings further illuminated the significant combined influence of two easily manipulable agronomic factors accessible to farmers worldwide seeking to increase saffron yields through data-driven production advances.

## Materials and methods

A factorial experiment under a completely randomized design (CRD) was conducted to evaluate the effect of saffron corm size and depth on its yield. Three planting depths 10cm, 15cm, and 20cm along with three corm sizes 05-10g (average weight;8.3g), 10.1-15g (average weight;12.8g), 15.1-20g (average weight;18.2g) were used. A soil mixture was prepared by thoroughly mixing equal ratios of clay loam soil, organic compost (grass residues and cow manure), and leaf litter (mainly *Syzygium cumini*). This mixed soil provided appropriate aeration, drainage, and nutrient content for saffron growth. The experiment was conducted after getting proper permission in a semi-arid climate at the research fields of the Department of Botany, The Islamia University of Bahawalpur, Pakistan during 2020–2021.

### Plant material

Fifteen uniform-sized corms for each category were obtained from a commercial farm in Quetta, Pakistan. The collection of plant material adhered to both local and national guidelines. All plant materials used were cultivated and not wild or endangered. The corms were examined for physical damage, rotting, or disease symptoms before experimental use. Plants were grown in cylindrical growing bags made of non-woven polypropylene fabric. Bags were 12 inches wide x 36 inches high (30.48 cm x 91.44 cm) with a volume of approximately 20 gallons (75 L). The bottom of the bags had pores for drainage. Given that the experiment was conducted in grow bags, field conditions were not applicable. However, the chemical and physical properties of the soil used in grow bags are given in Table 1. Corm quality assessment relied on visual inspection, with selections made based on the absence of visible damage or disease symptoms.

**Table 1:** Physiochemical properties of the soil used in the study.

Properties	Value
EC mS(cm^-1^)	0.69
pH	8.1
Organic matter (%)	1.00
Available phosphorus (mgKg^-1^)	2.45
Available potassium ((mgKg^-1^)	245
Saturation percentage	26
Gypsum required (ton/acre6)	Normal
Zn (ppm)	1.87
Cu (ppm)	0.10
Fe (ppm)	2.46
Mn(ppm)	1.12
B (ppm)	0.83
Remarks	Normal

### Sowing and hoeing

Grow bags, made of non-woven fabric, (imported from China) were filled with soil. Corms were sown on 09 November 2020. Five corms of each weight class were planted in separate rows in each bag, with three bags per treatment combination. Distance between corms was 5 cm and rows were 25 cm apart. The outer plant rows were 20 cm from the bag walls. To restrict the growth of weeds, hoeing was done on 21 December 2020. The soil became hard over time over time so hoeing was done to loosen it. This eases the uptake of water and encourages plant growth. However, no chemical fertilizer was used during the study.

### Irrigation

Saffron does not need water year-round. It does not grow well in poorly drained and over-watered soils. The water requirement of saffron is quite low and grows well in wet but not puddled soil. First irrigation was applied to maintain the field capacity at the time sowing; next irrigation was applied according to the soil moisture contents at the interval of 10–15 days.

### Soil and climate characteristics

The study period experienced fluctuating average maximum and minimum monthly temperatures. In November, temperatures ranged from 28°C to 13°C, followed by a range of 20°C to 7°C in December, and 22°C to 12°C in January. The average monthly precipitation, measured in millimeters, showed variations throughout the study period: 1mm in November, 0.2mm in December, and 0.7mm in January. Regarding monthly average humidity percentages, the levels fluctuated as follows: 52% in November, 58% in December, and 66% in January. The physiochemical properties of the soil are shown in Table 1.

### Data collection

#### Morphological parameters

The percentage of corms that emerged, number of leaves per plant, shoot length (length in cm of the tallest stolon), and number of stolons per plant were noted. The emergence percentage was calculated by dividing the number of emerged corms by the total number of corms sown for each treatment [14].

#### Agronomic parameters

Destructive root sampling was conducted 6 weeks after planting. Three plants per treatment were carefully extracted to retain intact root systems. Fresh mass of shoot, daughter corms, and entire plant as well as dry mass of shoot, corms, and entire plant were noted. For fresh weights, plants were washed, blotted dry, and weighed immediately after extraction. For dry weights, samples were oven-dried at 80°C until a constant weight was achieved. All weights were measured using a digital analytical balance (A&D CB600).

### Statistical analysis

Data was analyzed using Statistix 8.1 software. ANOVA (Analysis of Variance) was performed to compare different treatments and later mean separation was performed with Tukey’s HSD test. For drawing plots, Origin (2024) software.

## Results and discussion

### Emergence percentage

Data regarding emergence percentage is shown in Table 2. Results showed that sowing depth significantly affected the emergence of saffron. The corms sown under 15cm depth showed 100% emergence regardless of corm size (either 05-10g, 10.1-15g, 15.1-20g) followed by 10cm depth corms. Minimum emergence % was observed in corms sown under 20cm depth. These results align with the findings of Andabjadid *et al*. [15]. Our findings are also consistent with the findings of Yildirim *et al*. [16]. They examined saffron corms of varying sizes (large, medium, small) planted at either 5cm or 15cm depth. At 5cm depth, the number of emerged corms did not significantly differ by size. However, at 15cm depth, significant differences occurred based on corm size. Large corms showed the highest emergence rate while medium had the lowest. This could be attributed to the greater energy reserves and physiological vigor typically associated with larger corms. Larger corms often possess more stored nutrients and resources, facilitating robust early growth and emergence compared to smaller counterparts.

**Table 2:** Percentage emergence of *Crocus sativus* at Bahawalpur.

Depth (cm)	Size (g)	Percentage emergence
10	05–10	80 ^a^
	10.1–15	100 ^a^
	15.1–20	100 ^a^
15	05–10	100 ^a^
	10.1–15	100 ^a^
	15.1–20	100 ^a^
20	05–10	60 ^c^
	10.1–15	100 ^a^
	15.1–20	80 ^b^

### Corm dry weight

**Fig 1:**
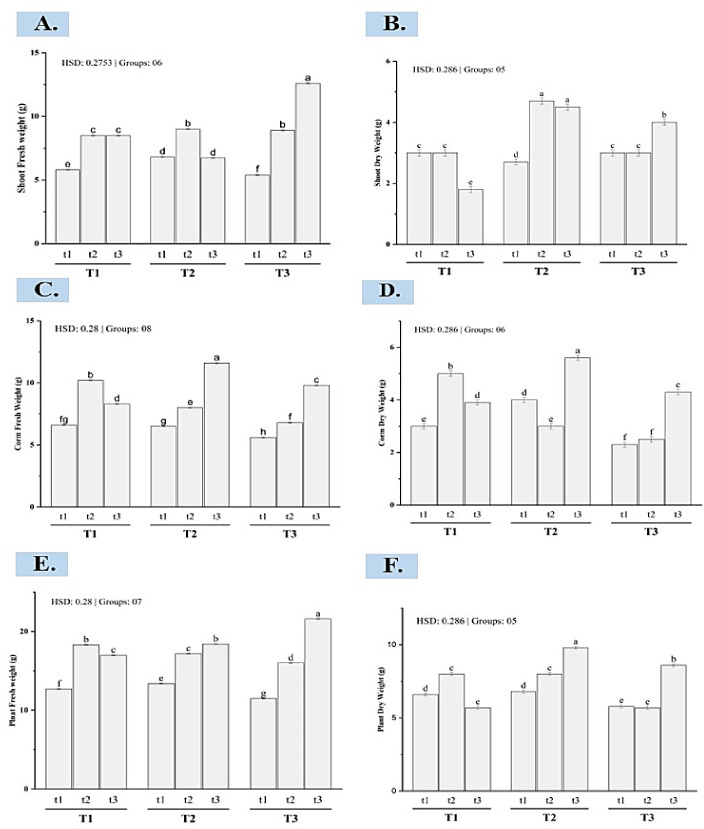
Bar plot of multiple comparisons of means using Tukey’s honest significant difference (HSD) test. (A) Shoot Fresh weight (g), (B) Shoot Dry weight(g), (C) Corm Fresh weight (g), (D) Corm Dry weight (g), (E) Plant Fresh weight (g), (F) Plant Dry weight (g). Means with different letters are significantly different at alpha 0.05 from each other. T1, T2, and T3 represent sowing depths as 10cm, 15cm, and 20cm while t1, t2, and t3 represent corm sizes as 05-10g, 10.1-15g, 15.1-20g respectively.

The results from the ANOVA analysis show that both the depth at which the plants were planted and the initial size of the corm has a significant impact on the dry weight of the corm. This impact is highly significant (p < 0.001) for both factors. There is also a significant interaction between these two factors (p < 0.001). Further analysis using Tukey’s post-hoc comparisons elucidates the nature of this interaction effect (Fig 1D). Specifically, the effect of initial corm size on eventual dry-weight biomass production depends substantially on planting depth. At the shallowest depth tested (T1), corms of intermediate size (t2) achieved greater dry weight than either smaller (t1) or larger (t3) corms. However, at moderate depth T2, the largest-sized corms (t3) produced significantly higher dry biomass than smaller sizes t1 and t2 by harvest. Finally, at the greatest planting depth (T3), there were significant differences in dry weight among all three corm sizes by the conclusion of the experiment, with the rank order of t3 > t2 > t1 from highest to lowest mean corm mass.

Larger corm size generally confers an advantage in terms of greater resultant biomass, but this effect interacts with planting depth such that the largest corms show the greatest benefit when sown deeply, while intermediate sizes are optimal for shallow planting depths. This is likely due to greater energy reserves and physiological vigor typically associated with larger corms and enhanced access to soil nutrients and moisture when sown deeply. Conversely, intermediate-sized corms exhibit optimal performance when sown in shallower depths, potentially capitalizing on more accessible resources closer to the surface [9, 7]. These findings align with the conclusions drawn by Ahmed *et al*. [17], who similarly reported a significant influence of corm size (8-11g) on the dry weight of corm, corroborating the importance of this factor in saffron cultivation. Furthermore, our results are also consistent with the trends identified by Sharifi *et al*. [8], reinforcing the understanding that corm size (8±1 g) and planting depth (15±2 cm) significantly contribute to the variability in saffron emergence and growth. Our findings also align with Mollafilabi *et al*. [13] who studied three different weights of corm (6–8, 8–10, and >10 g) and found all the traits to be significantly affected by increasing corm size. Banhangi *et al*. [7] have also observed that increasing sowing depth from 10 to 20cm reduces total dry matter.

### Corm fresh weight

The analysis of corm fresh weight reveals significant main effects of both initial corm size and planting depth, as well as a significant interaction between these two factors (p < 0.001 for all effects). Examining the post-hoc comparisons shows that the interaction arises from differences in the optimal corm size across depths (Fig 1C). Specifically, the largest corm size (t3, 15.1-20g) produced the greatest fresh-weight biomass at the deepest planting depth of 20cm (T3), while intermediate-sized corms (t2, 10.1-15g) were superior at the shallowest 10cm depth (T1). However, at the moderate 15cm depth (T2), the smallest corms (t1, 5-10g) performed equivalently to the intermediate size.

Larger corms generally confer an advantage in terms of greater final fresh weight, but substantial interactions with sowing depth must be considered to optimize propagule size. Planting at maximal depth favored the biggest corms, whereas shallow depths benefited from intermediate sizes. The preference for maximal planting depth by larger corms likely stems from their greater reserves, enabling them to access deeper soil nutrients and moisture effectively. Conversely, intermediate-sized corms thrive in shallower depths, where they can efficiently utilize available resources for optimal growth and development [7, 9, 16]. These findings align with the results of Mollafilabi *et al*. [18]. They observed a positive correlation among all agronomic parameters and corm size (6–8, 8–10, and more than 10 g). The findings of our research are also consistent with Yildirim *et al*. [16]. They observed more fresh weight of corms at 15cm depth. However, regarding the sowing depth of corm, our findings are contrary to Koocheki and Seyyedi [10]. They observed that surface planting (10cm) produced more fresh weight in corms as compared to the deepest sown (20cm) corm. Further delineation of these interacting effects would assist saffron producers in selecting ideal initial corm sizes tailored to their typical planting depths for superior productivity.

### Shoot fresh weight

The ANOVA results for shoot fresh weight indicate a significant effect of both corm size and planting depth as well as their interaction (p < 0.001). The post-hoc comparisons (Fig 1A) show that the largest corms (t3) maximized shoot fresh biomass production when sown at the greatest depth (T3). However, at T1 and T2 depths, intermediate-sized corms (t2) optimized shoot productivity, significantly outperforming other size x-depth combinations. Meanwhile, small corms (t1) generated the lowest shoot fresh weights across all depths. These findings align with the results of Mollafilabi *et al*. [18]. They observed that large-sized mother corms (>10g) improved all growth parameters. Yildirim *et al*. [16] documented that shallow sowing results in better growth as compared to deep sowing. Deep planting provides the greatest shoot biomass when exploiting large initial propagules, but intermediate corm sizes are ideal for typical shallower sowing conditions. The findings from our study hold practical implications for saffron cultivation strategies in the field. For instance, in areas where deep planting is feasible and desired, such as regions with ample soil depth or where moisture retention is crucial, growers can capitalize on the superior shoot biomass production associated with large initial propagules. Conversely, in shallower sowing conditions common in many saffron-growing regions, selecting intermediate corm sizes can optimize resource utilization and promote robust growth.

### Shoot dry weight

The analysis of shoot dry weight indicates significant main effects of both initial corm size and planting depth, as well as a significant interaction between the two factors (p < 0.001 for all effects). Examining the post-hoc comparisons shows that intermediate-sized corms (t2) optimize shoot dry biomass production when sown at moderate depth (T2), significantly outperforming all other corm sizes x depth combinations (Fig 1B). Meanwhile, the largest corms (t3) generated the greatest shoot mass at deep planting (T3) but performed worst with shallow sowing (T1). Small corms (t1) showed an intermediate response across depths.

Intermediate corm sizes favor shoot productivity when moderately planted, whereas deep sowing benefits most from large initial propagules. Shallow planting depths confer little advantage to any corm size for shoot biomass. Kumar *et al*. [9] have also observed that a planting depth of 7-10cm produces more dry weight than deep sowing. This significant crossover interaction highlights the need to synchronize planting depth and corm sizes to maximize shoot yields. By strategically aligning planting practices with specific production goals, saffron producers can enhance overall yield outcomes. For instance, emphasizing rapid foliage generation through the selection of intermediate-sized corms planted at typical depths may lead to earlier harvests and increased short-term yields. Conversely, prioritizing long-term biomass accumulation by exploiting larger corms planted deeply can potentially result in higher yields over extended cultivation periods, thus offering a strategic approach to maximizing plant commercial yields in saffron production.

### Plant dry weight

The analysis of total plant dry weight indicates significant main effects of both initial corm size and planting depth, as well as a significant interaction between these factors (p < 0.001 for all effects). The post-hoc test (Fig 1F) reveals that the largest corm size (t3) produced the greatest plant biomass when sown at the deepest depth (T3). However, at shallow planting depths, small (t1) and intermediate (t2) sized corms performed equivalently to the largest size (t3). Meanwhile, intermediate corms (t2) optimized dry weight at the moderate depth (T2), significantly outperforming all other sizes x depth combinations. Mollafilabi *et al*. [18] have also observed similar findings with larger corm size (>10g) producing more biomass. Considering the saffron plant, the majority of its biomass is made up of saffron corms. To boost the overall dry matter of the plant, it’s essential to enhance the agronomic conditions for corm growth. An interesting finding by Gresta *et al*. [19] reveals that as planting depth increases, the propagation power of corms decreases. This is because deeper planting conditions lead to higher soil moisture and lower soil temperature, resulting in a reduction in the weight of daughter corms. Kumar *et al*. [9] further supported this notion by noting that corms planted at shallower depths (7.5–10 cm) show greater weight accumulation in the initial stages compared to those planted deeper (12.5–15 cm).

### Plant fresh weight

The analysis of total plant fresh weight shows highly significant main effects of initial corm size and planting depth, as well as a significant interaction between the two factors (p < 0.001 for all effects). Further examination of the post-hoc comparisons indicates that the largest corm size (t3) maximized plant fresh biomass production at the greatest planting depth (T3) (Fig 1E). However, at shallow and moderate depths, small (t1) and intermediate (t2)-sized corms performed statistically similarly to the largest corms. Meanwhile, intermediate corms optimized plant fresh weight when sown at the middle depth (T2), significantly exceeding all other size x-depth combinations.

The deepest planting depth consistently favored large initial propagules for the greatest productivity. But in shallow or moderate conditions, small and intermediate-sized corms proved comparable, with intermediate sizes optimal at middle depths. As such, maximum planting depth allows large corm exploitation for the greatest yields, yet shallower sowing provides more flexibility in size selection without forfeiting productivity. Mollafilabi *et al*. [18] have also observed similar findings with larger corm size (>10g) producing more biomass. Our research findings also align with Yildirim *et al*. [16], who also discovered that plants had a higher fresh weight at a depth of 15cm. However, our results differ from those of Koocheki and Seyyedi, [10], who observed that planting the corm at the surface (10cm) resulted in greater fresh weight compared to deep sowing (20cm). This suggests that the optimal sowing depth for achieving maximum fresh weight may vary depending on the specific conditions.

### Number of daughter corms

**Fig 2:**
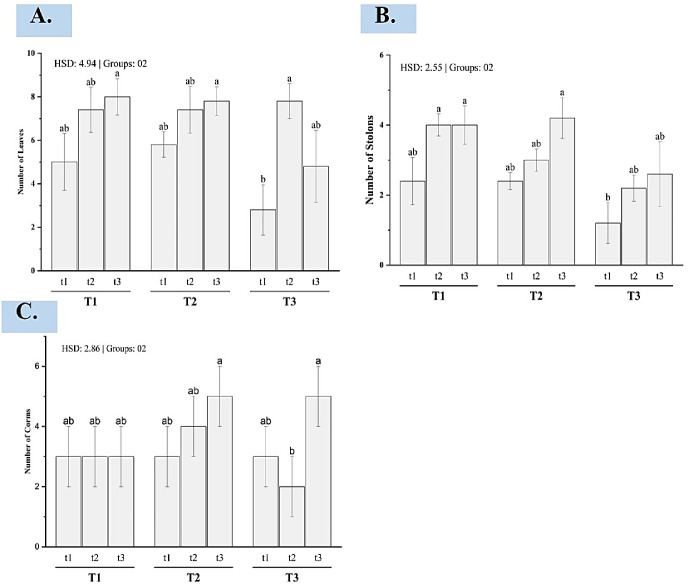
Bar plot of multiple comparison of means using Tukey’s honest significant difference test. (A) number of leaves, (B) number of stolons, (C) number of corms. Means with different letters are significantly different at alpha 0.05 from each other. T1, T2, and T3 represent sowing depths as 10cm, 15cm, and 20cm while t1, t2, and t3 represent corm sizes as 05-10g, 10.1-15g, and 15.1-20g respectively. Further examination of the post-hoc comparisons (Fig 2A) indicates that large (t3) and intermediate (t2) sized corms produced significantly more leaves compared to small corms (t1), averaged across planting depths. Depth itself did not significantly impact leaf counts. While greater initial corm size enhanced leaf productivity, planting depth had negligible effects either independently or in combination with size. The lack of significant depth or interaction effects suggests leaf initiation rates depend predominantly on initial propagule size alone. Therefore, saffron producers aiming to maximize foliage numbers should focus efforts on sowing the largest viable corms, rather than precision depth matching.

The analysis of the number of corms produced reveals a significant main effect of initial corm size (p = 0.0152). However, there was no significant impact of planting depth (p = 0.1256) or the corm size x depth interaction (p = 0.0949) on resultant corm counts. Examining the posthoc test shows that large corms (t3, 15.1-20g) generated significantly higher daughter corm numbers on average compared to small corms (t2, 5-10g) across all planting depths, with intermediate sizes (t2) being statistically similar to both extremes (Fig 2C). This suggests that corm size plays a crucial role in determining reproductive success in saffron cultivation. Larger corms likely possess greater reserves, promoting enhanced daughter corm production.

Our findings align with previous work indicating that larger saffron mother corms produce more and heavier daughter corms in subsequent years [20, 21]. Specifically, we demonstrated significant positive relationships between initial corm size and resultant metrics including corm counts, fresh weight, and dry biomass. These results support the conclusions of [22], who compared small and large corms and found greater daughter production and weights from larger initial propagules. Similarly, Gresta *et al*. [23] showed that the magnitude of a mother corm directly enhances yields of progeny corms. These findings are consistent with the results of [15]. Khorramdel *et al*. [24] have also documented similar findings with a considerable increase (241%) in quantity and weight of daughter corms with increasing weight of mother corm. Furthermore, Ahmed *et al*. [17] have also observed a similar trend. While deeper sowing depths and greater initial propagule sizes enhanced metrics like corm fresh weight and dry biomass in this study, resultant daughter corm quantity appears primarily dictated by mother corm size alone. The lack of significant depth or interaction effects suggests that planting depth exerts minimal influence on multiplication rates. Therefore, saffron producers emphasizing rapid stand proliferation over biomass would benefit most from large initial corm stock, regardless of their typical sowing depths.

### Numbers of stolon

The analysis of the numbers of stolon produced shows significant main effects of both initial corm size and planting depth (p = 0.0035 and p = 0.0052, respectively). However, the interaction between corm size and depth was non-significant (p = 0.8282). Examining the post-hoc test (Fig 2B) reveals that large (t3) and intermediate (t2) sized corms generated greater ramification than small corms (t1) across planting depths. Meanwhile, shallow (T1) and moderate (T2) depths enhanced ramification compared to deep planting (T3), averaged across corm sizes. Larger initial propagule size and shallower sowing depth independently increased saffron ramification. However, the lack of significant interaction suggests their effects are additive, rather than synergistic or antagonistic. Growers targeting prolific ramification should utilize the largest viable corm sizes planted at optimal shallow to moderate depths.

### Number of leaves

The analysis of leaf number reveals a significant main effect of initial corm size (p = 0.0036) on resultant foliage quantity. However, the main effect of planting depth and the corm size x depth interaction was non-significant (p > 0.05).

The practical application of the study’s conclusions lies in the optimization of saffron cultivation practices. Farmers can benefit from the findings by selecting corm sizes based on their desired outcomes, with larger corms generally leading to increased biomass. Additionally, planting depth plays a crucial role, as the study highlights that a 15cm depth results in the highest emergence rates. Tailoring corm selection and planting depth to specific conditions can enhance saffron production, with intermediate-sized corms performing well at moderate depths and larger corms thriving in deeper conditions. The positive correlation between initial corm size and daughter corm production emphasizes the importance of selecting larger corms for higher yields. Farmers are encouraged to consider both factors, corm size and planting depth, for optimal results, providing evidence-based guidelines to improve saffron cultivation practices and address challenges associated with declining global saffron production.

## Conclusions

In conclusion, our study systematically explored the interactive effects of different corm sizes and planting depths on saffron growth patterns and yields. The findings offer valuable insights into optimizing cultivation practices for enhanced saffron production. The results demonstrated that planting depth significantly influenced emergence percentages, with 15cm depth showing the highest emergence rates. Furthermore, the study revealed a complex interaction between corm size and planting depth in affecting corm dry weight. Larger corm sizes generally resulted in greater biomass, but this effect varied with planting depth, emphasizing the importance of coordinated management practices. Notably, the analysis of various parameters such as shoot fresh weight, shoot dry weight, plant fresh weight, and plant dry weight consistently highlighted the interplay between corm size and planting depth. Intermediate-sized corms often showed optimal performance at moderate depths, while larger corms excelled in deeper planting conditions. The study also confirmed the positive correlation between initial corm size and daughter corm production, indicating the significance of selecting larger corms for higher yields. Interestingly, the number of stolons and leaves was influenced by both corm size and planting depth, with larger corm sizes favoring increased ramification and leaf production. However, planting depth independently impacted these parameters, emphasizing the need for considering both factors for desired outcomes. These findings contribute to the existing body of knowledge on saffron cultivation by providing evidence-based guidelines for farmers seeking to improve yields. The practical implications of our study underscore the importance of tailoring corm selection and planting depth to specific conditions, allowing for a more nuanced and effective approach to saffron cultivation. As saffron continues to be a high-value spice with economic significance, adopting these data-driven insights can play a pivotal role in addressing the challenges associated with declining global saffron production.
