# Expression of Concern: Revealing the dynamics of saffron growth: Optimizing corm size and planting depth for increased yield synergies

**DOI:** 10.1371/journal.pone.0316036

**Published:** 2024-12-13

**Authors:** The PLOS ONE Editors

Contrary to the declaration in the Data Availability statement, the original raw data files supporting the article’s results were not provided with the article, and the authors have not provided these data following editorial request.

Since the original raw data files supporting the article’s results have not been made available, this article [1] does not comply with the PLOS Data Availability policy. The *PLOS ONE* Editors therefore issue this Expression of Concern.

After this article [1] was published, author MI contacted the journal to state that they were unaware of the article’s submission and did not consent to either publication of this work or the authorship contributions listed in the article. The corresponding author disputed this statement, stating that MI was informed and agreed to authorship. As of the time of publication of this notice PLOS has not received institutional input pertaining to the authorship, data ownership, or author contributions to this article.
